# Differential Expression of miRNAs in *Brassica napus* Root following Infection with *Plasmodiophora brassicae*

**DOI:** 10.1371/journal.pone.0086648

**Published:** 2014-01-31

**Authors:** Shiv S. Verma, Muhammad H. Rahman, Michael K. Deyholos, Urmila Basu, Nat N. V. Kav

**Affiliations:** 1 Department of Agricultural, Food and Nutritional Science, University of Alberta, Edmonton, Alberta, Canada; 2 Department of Biological Sciences, University of Alberta, Edmonton, Alberta, Canada; Nanjing Agricultural University, China

## Abstract

Canola (oilseed rape, *Brassica napus* L.) is susceptible to infection by the biotrophic protist *Plasmodiophora brassicae*, the causal agent of clubroot. To understand the roles of microRNAs (miRNAs) during the post-transcriptional regulation of disease initiation and progression, we have characterized the changes in miRNA expression profiles in canola roots during clubroot disease development and have compared these to uninfected roots. Two different stages of clubroot development were targeted in this miRNA profiling study: an early time of 10-dpi for disease initiation and a later 20-dpi, by which time the pathogen had colonized the roots (as evident by visible gall formation and histological observations). *P. brassicae* responsive miRNAs were identified and validated by qRT-PCR of miRNAs and the subsequent validation of the target mRNAs through starBase degradome analysis, and through 5′ RLM-RACE. This study identifies putative miRNA-regulated genes with roles during clubroot disease initiation and development. Putative target genes identified in this study included: transcription factors (TFs), hormone-related genes, as well as genes associated with plant stress response regulation such as cytokinin, auxin/ethylene response elements. The results of our study may assist in elucidating the role of miRNAs in post-transcriptional regulation of target genes during disease development and may contribute to the development of strategies to engineer durable resistance to this important phytopathogen.

## Introduction

Plant pathogens are devastating biological factors that adversely affect plant growth and development [1] Various plant pathogen infections can cause up to 30% yield losses in many crops [2]. Infection of the Brassicaceae family with the obligate biotrophic pathogen *Plasmodiophora brassicae* Woronin, a cercozoan protist belonging to the class phytomyxea, results in the development of root galls (clubroots) and consequent stunting of plants [3], [4]. Clubroot disease has been reported in more than 60 countries resulting in overall reduction in the yield of canola by about 10–15% [5]. In Alberta, Canada, approximately 94% of plants were observed to be affected in most infected fields, resulting in an estimated yield loss of about 30% [6]. Several potential management strategies can be used to control *P. brassicae* infestation on canola and other cruciferous crops. For example, biocontrol agents (*Bacillus subtilis* and *Gliocladium catenulatum*) and fungicides (Fluazinam and Cyazofamid) have been used to lower disease severity [7]. However, a longer-term solution would be the development of durable resistance to this pathogen through classical breeding or by genetic modification, which necessitates the identification of targets for genetic manipulation.

In previous studies, genotypes of the *Brassica* species with resistance to broad-spectrum pathotypes of *P. brassicae* were identified [8], and these were classified as pathotype-dependent resistance or race-specific [9]. Recently, [10] ten *P. brassicae* genes were identified that are expressed during the infection of Chinese cabbage (*B. rapa* subsp. pekinensis). These genes were identical to those previously observed to be modulated during infection of *Arabidopsis* plants with *P. brassicae* [11]. Also, a group of scientists from Japan recently identified clubroot resistance genes (*Crr1a*, *CRa* and *CRb*) through map-based cloning, which confer resistance against *P. brassicae* (pathotype group 3) in *B. rapa* [12], [13], [14]. Moreover, transcriptomic [15] and proteomic analyses [16], [17] have previously indicated the involvement of hormone regulation during clubroot infection. Furthermore, the plant hormones auxin and cytokinin have also been implicated in development of root galls in cruciferous crops [18], [19], [20], [21]. Despite these reports, information on regulatory mechanisms involving TFs and changes in post-transcriptional regulation at miRNA level during *P. brassicae* infection or club formation is lacking.

MicroRNAs are a highly conserved class of small noncoding RNAs that regulate gene expression by post-transcriptional repression [22], [23]. Emerging evidence indicates that hosts’ endogenous small RNAs represent an important mechanism of control in plant immune responses [24] and hormone signaling at the time of stress [25]. For example, ath-miR160 and ath-miR167 are involved in pathogenesis and target the auxin-response-factor (ARF) [26], [27]. Another microRNA, ath-miR164, has been implicated in auxin homeostasis and lateral root development [28], which may have a bearing on clubroot development. Therefore, it is conceivable that miRNAs may be involved in mediating important plant processes following infection with *P. brassicae*, which results in the development of root galls, leading to stunting and decreased productivity.

Most methods for identification of biotic responsive miRNAs are not sensitive enough to identify miRNAs expressed at low levels. Recently, microarrays of miRNAs have become available for high-throughput global analysis of miRNAs expression patterns in response to pathogens [29]. This valuable tool has been further exploited in a number of plants to study the miRNA: mRNA interactions in tomato, rice and *Arabidopsis* [30], [31], [32] which may lead to the identification of miRNAs that are involved in plant responses to specific stresses, including *P. brassicae* challenge.

In this study, we report the miRNA expression profiles in roots of *B. napus* plants in response to challenge with clubroot pathogen infection. These results are discussed in context with hormone homeostasis, and the regulation of TFs during disease development and progression.

## Results and Discussion

### Phenotypic Changes in Roots with Response to *P. brassicae*

**Figure 1:**
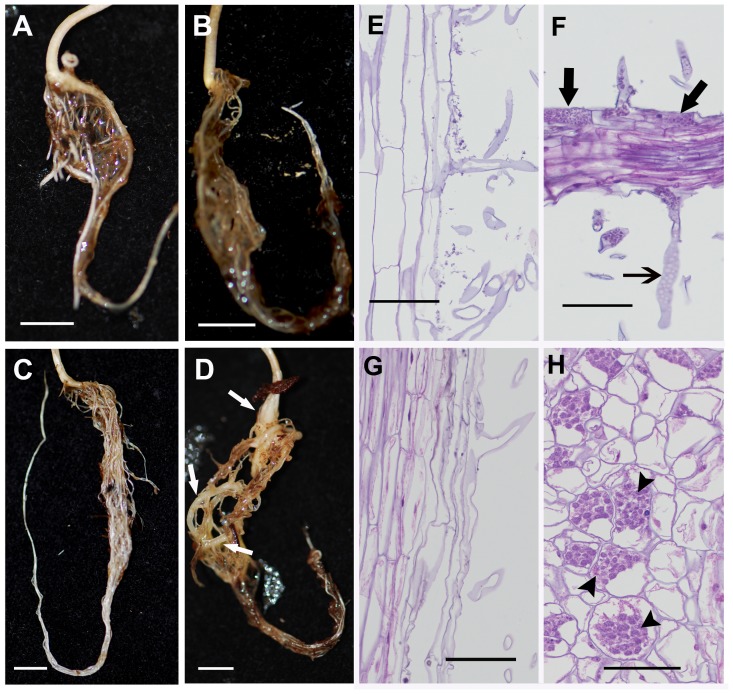
Morphology if 10-day old healthy (A), and clubroot-infected (B), and 20 day old uninfected (C), and clubroot infected (D) *B. napus* roots showing gall formation in the latter (open arrows) due to *P. brassicae* infection. Histopathological analysis indicates the presence of evacuated zoosporangia (open arrow) in the root hair and primary plasmodia (closed arrows) in epidermal cells indicating infection (F) compared to uninoculated controls (E). At 20 dpi, the cortical cells of infected tissue show the presence of numerous secondary plasmodium (arrowheads) in the cortical cells (H) compared to the control (G). Bars represent 5 mm for Figures. A–D and 100 µm for Figures. E–H.

At the earlier time point of 10 day post inoculation (dpi), compared to the uninoculated controls (Figure 1A), no obvious swelling of the pathogen treated roots was observed (Figure 1B). However, at 20 dpi, the inoculated roots exhibited swelling (Figure 1D, arrows), which is a typical characteristic of the clubroot infection. The uninfected roots of the corresponding stage, on the other hand, exhibited typical long and fibrous root system (Figure 1C). Although there was no visible symptom development at 10 dpi, microscopic investigation showed the colonization of the roots by pathogen (Figure 1F). While uninoculated roots were elongated and highly vacuolated (Figure 1E), the inoculated roots exhibited the presence of evacuated zoosporangia (Figure 1F, open arrow) similar to the observations with turnip at day 12 (Asano et al. 1999), as well as primary plasmodium with zoospores (Figure 1F, closed arrows). Therefore, despite the absence of visible morphological symptoms of clubroot infection at 10 dpi, histological observations indicated that the *Plasmodiophora* infection and colonization has indeed begun at this time point. At 20 dpi, the cortical cells showed the evidence of hypertrophy, along with the presence of secondary plasmodium (Figure 1H, arrowheads) compared to elongated, vacuolated cells of the uninfected control (Figure 1G). The increased hypertrophy in clubroot infected *Arabidopsis* root cells has been reported to be responsible for gall formation [33].

### *P. brassicae* Responsive miRNA

**Figure 2:**
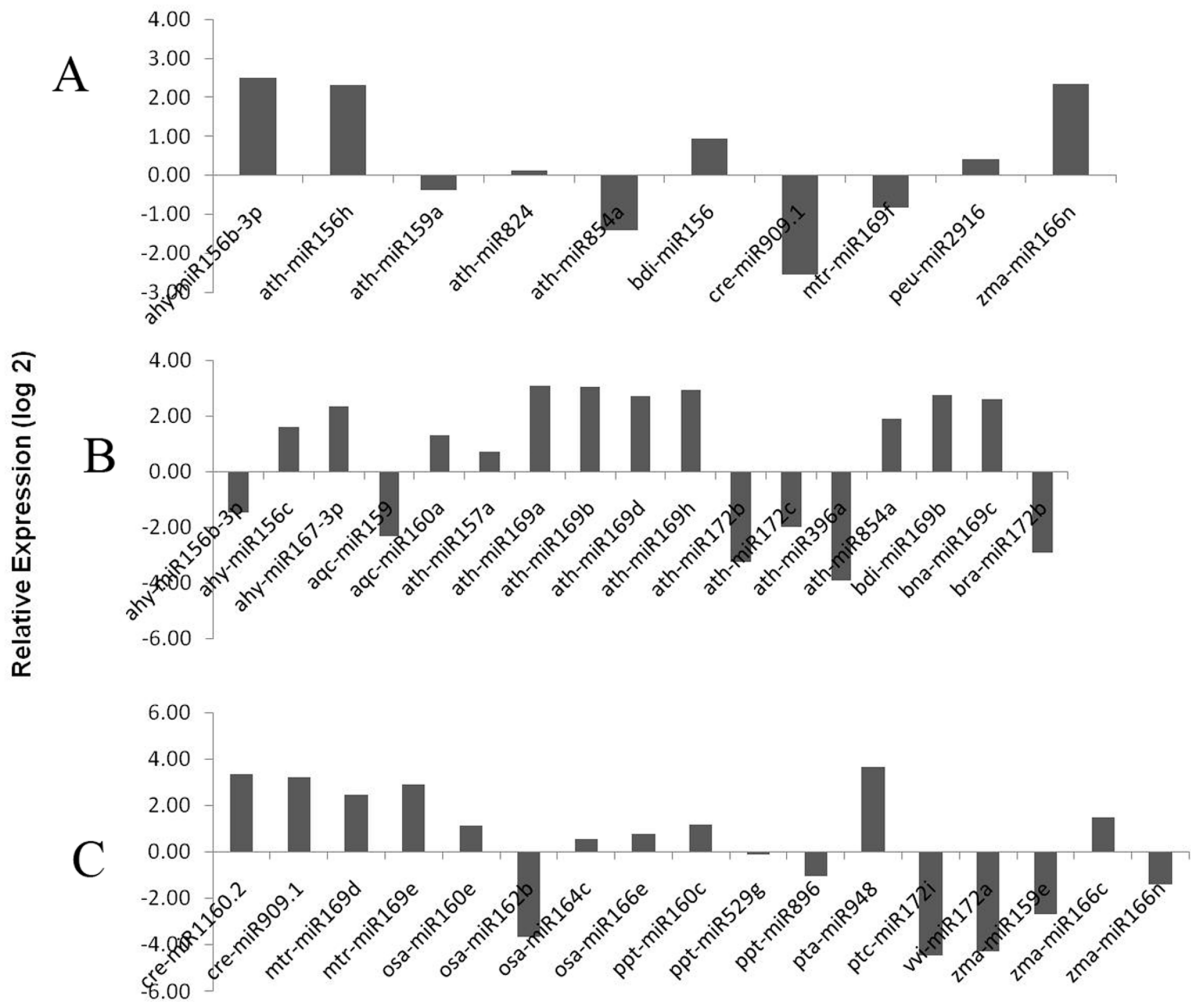
miRNA-microarray expression of *P. brassicae* responsive miRNAs exhibiting differential expression at 10- (A) and 20- dpi (B, C) following pathogen infection.

The differential expression of *B. napus* miRNAs following inoculation with the biotrophic protist *P. brassicae* was compared to a mock sample (untreated) at two time points (10 and 20 dpi) of clubroot disease development. In the miRNA based microarray, we observed that ten miRNAs were differentially expressed at the earlier time point (10 dpi; Figure 2A) and 34 miRNA showed differential expression at the later time point (20 dpi; Figure 2B, C) in response to *P. brassicae* infection. Among those miRNAs that were modulated at 10 dpi, most of them (bdi-miR156, ath-miR156h, ath-miR824 and peu-miR2916) showed an increase in abundance, while the level of mtr-miR169f decreased (Figure 2A). On the other hand, at 20 dpi, 21 miRNAs were increased in abundance, whereas 13 miRNAs exhibited decreased abundance (Figure 2B, C). Interestingly, the miRNAs ahy-miR156b-3p and zma-miR166n increased at 10 dpi, but decreased in abundance at 20 dpi; whereas levels of ath-miR854a and cre-miR909.1 decreased at 10 dpi and increased at 20 dpi (Figure 2, S1, S2).

Among the differentially expressed *P. brassicae* miRNAs, ath-miR156 increased during the early time point (10 dpi) and decreased at the later stage (20 dpi). This miRNA has been previously reported to be involved in hormone homeostasis (abscisic acid signaling, gibberellin response), in mediating responses to abiotic stresses [34], [35] and is also induced by Turnip Mosaic Virus (TuMV) infection in transgenic *Arabidopsis* plants [36], [37]. The zma-miR166, which is also increased in abundance at 10 dpi (Figure 2A), has previously been shown to be involved in the regulation of class-III homeodomain leucine zipper (*HD-ZIP III*) genes [38]. The *HD-ZIP III* TFs have also been implicated in lateral root development of *A. thaliana* [39]. The expression of zma-miR166n increased at 10 dpi and decreased at 20 dpi. Interestingly, the over-expression of mtr-miR166 has been previously shown to reduce the number of symbiotic nodules and lateral roots in *Medicago truncatula* [40]. Our results suggest that the miRNAs, which have been implicated in root development and/or in mediating plant responses to pathogens, are modulated during clubroot development.

Interestingly, we identified eight miR169s, all of which showed increased expression due to infection of *B. napus* with *P. brassicae* (Fig. 2b, c) at 20 dpi. However, alignment of these mature miRNA sequences exhibited at least 18 of the 21 bases being conserved, with only between one and three bases at the termini being dissimilar (data not shown), indicating their widespread and highly conserved nature among plant species. The conservation of miRNA sequences between different plant species, with variations of one or a few base pairs, has previously been reported [25]. The expression of ath-miR169 was found to be increase at 20 dpi. A similar increase in abundance of the ath-miR169 has been reported following pathogen attack in the *Arabidopsis* [24] as well as in response to osmotic stress in rice [31], [41]. However, the abundance of ath-miR172 decreased at 20 dpi following *P. brassicae* infection and was not detectable at 10 dpi. It has been previously reported that the abundance of ath-miR172 was decreased due to UV-B radiation in *Arabidopsis* [42], and in response to viral infection in tomato [30]. The levels of aqc, osa, ppt-miR160 increased at 20 dpi and were not detectable at 10 dpi. These miRNAs have been shown to be increased in pine stems with response to the rust fungus [43] but increased in *Arabidopsis* due to bacterial infection [44]. Increased expression of ath- miR160 in response to hypoxia in *Arabidopsis* [45] and maize [46] possibly indicates the involvement of the miRNAs in both abiotic and biotic stress response.

Our observations also revealed that ath-miR396 was differentially expressed during the disease progression and decreased at 20 dpi (Figure 2C). The expression of tae-miR396 has been reported to be decreased in wheat (*Triticum aestivum*) following powdery mildew infection [47]. Interestingly, the expression of cre-miR909 decreased at 10 dpi, while at the later stage of disease progression (20 dpi), it was increased. The role of cre-miR909 in relation to its involvement in stress response has not been reported yet. In the current study, miRNA microarray analysis led to the identification of several pathogen (*P. brassicae*) responsive miRNAs in *B. napus*. Interestingly, most of these miRNAs were decreased in abundance, which suggests that expression of their target genes could be enhanced in response to *P. brassicae* infection.

### miRNA-mRNA Target Prediction

Potential miRNA targets were identified using degradome analysis [48]. In our study, many of the predicted target genes of miRNAs that were differentially expressed during clubroot disease progression belonged to various families of TFs (Table 1). In most of the cases they showed G:U base pair or a single nucleotide mismatch and, indeed, a G:U base pair with 7–8 mer seed site has been reported to cause down-regulation of target genes [23]. Consistent with the previous observations [49], single mismatches within the miRNA:mRNA interaction target position (2–8) were shown to strongly reduce expression of target genes (Table 2). However, in some miRNA:mRNA interactions, two simultaneous mismatches at the 3′ region had only little or small effect on target suppression [50].

**Table 1:** List of miRNAs that exhibited modulation in their expression following infection by *P. brassicae*, their possible targets, and their annotated biological functions.

miRNAs	Target gene	Target Function*
ath-miR156h	SBP-Like genes	Auxin signalling; from germination to mature seeds; inflorescence
ath-miR159	MYB33	hormone signalling during stress response
aqc-miR160a	ARF	Involved in regulating early auxin response genes
zma-miR166n	HD-ZIPIII	Polarity of Leaf
osa-miR164c	NAC	Involved in shoot apical meristem formation and auxin-mediated lateral root formation.
mtr-miR169d	NFY	Involved in hormone homeostasis during stress
vvi-miR172a	AP2 like	Involved in regulation of TFs during the pathogen response
ath-miR824	MADS box	Root development, trichome and guard cells
cre-miR909	LEA/Auxin repressed like	Auxin signalling
ath-miR396	TIR1/GRF	Adaptive response to stress
peu-miR2916	F-box protein	Involved in pathogen induced response; Aux/IAA signalling

* The targets were predicted employing starBase - degradome analysis software as indicated in “Materials and Methods”.

**Table 2:** Possible interactions between identified miRNAs showing modulation and their predicted targets as obtained through starBase Degradome analysis.

miRNA		miRNA:mRNA interaction
	miRNA_3′ CACGAGAGAAAGAAGACAGU	
**ath-miR156h**	|||||||||.||||||||||	
	target_5′ GUGCUCUCUCUCUUCUGUCA	
	miRNA_3′ AUCUCGAGGGAAGUUAGGUUU	
**ath-miR159**	||||||.||||||||.|||||	
	target_5′ UAGAGCCCCCUUCAAACCAAA	
	miRNA_3′ CACGAGAGAUAGAAGACAGUU	
**ath-miR157a**	|||||||||.|||||||||||	
	target_5′ GUGCUCUCUCUCUUCUGUCAA	
	miRNA_3′ GGCCGUUCAGUAGGAACCGAA	
**ath-miR169b**	|||||||.||||o||||||||	
	target_5′ CCGGCAAAUCAUUCUUGGCUU	
	miRNA_3′ GACGUCGUAGUAGUUCUAAGA	
**ath-miR172c**	||||||||||||||o||||||	
	target_5′ CUGCAGCAUCAUCAGGAUUCU	
	miRNA_3′ AGCCGUUCAGUAGGAACCGAC	
**ath-miR169a**	||||.|||.|||||||||||.	
	target_5′ UCGGAAAG-CAUCCUUGGCUC	
	miRNA_3′ GUGGUGGCCAAACUGGUCGU	
**cre-miR909.1**	|o|||||||||||o||||||	
	target_5′ CGCCACCGGCUUGAUCAGCA	
	miRNA_3′ GAGGAGGAGGGAUAGGAGUAG	
**ath-miR854a**	| |||||||||||||||||o||	
	target_5′ CUCCUCCUCCCUAUCCUCGUC	
	miRNA_3′ AGGGAAGAGUGUUUACCAGAU	
**ath-miR824**	| |o||||||||||||||||||	
	target_5′ UCUCUUCUCACAAAUGGUCUA	
	miRNA_3′ CACGAGAGAGAGAAGACAGU	
**bdi-miR156**	|||||.||||||||||||||	
	target_5′ AUGCUCCCUCUCUUCUGUCA	
	miRNA_3′ ACUAGCAGAAGCUCAGGGGU	
**peu-miR2916**	||||o||||||.||||||||	
	target_5′ UGAUUGUCUUCAAGUCCCCU	
	miRNA_3′ CCCUUACUUCGGACCAAGCU	
**osa-miR166e**	||.||||||||| ||||o|||	
	target_5′ GGCAAUGAAGCCGGGUUUGA	
	miRNA_3′ GGCCGUUCAGUAGGAACCGAA	
**mtr-miR169d**	|.|||||.||||o||||||||	
	target_5′ CAGGCAACUCAUUCUUGGCUU	
	miRNA_3′ CCGUAGGUCCCUCGGUCCGU	
**aqc-miR160a**	| | |.|||||||.|||||||||	
	target_5′ GUCCUUCAGGGAGUCAGGCA	
	miRNA_3′ CCGUUCAGUAGGAACCGAGU	
**ath-miR169d**	||||| |||||o|||||||||	
	target_5′ GGCAAAUCAUCUUUGGCUCA	
	miRNA_3′ GCCGUUCAGUAGGAACCGAU	
**bdi-miR169b**	o|||||.||||||||||||	
	target_5′ UGGCAAAUCAUCCUUGGCUU	
	miRNA_3′ ACGUCGUAGUAGUCCUAAGA	
**ptc-miR172i**	| |||o|||||||||||||||	
	target_5′ UCCAGUAUCAUCAGGAUUCU	
	miRNA_3′ CGUGCAUGGGACGAAGAGGU	
**osa-miR164c**	|| |||o||||||||||||||	
	target_5′ GCUCGUGCCCUGCUUCUCCA	
	miRNA_3′ CCACUUUGACGGUGUACUAGA	
**ahy-miR167-3p**	|| |||||||oo||||||||||	
	target_5′ GGAGAAACUGUUACAUGAUCA	
	miRNA_3′ CCGUACGUCCCUCGGUCCGC	
**ppt-miR160c**	|||||||||||||||||||.	
	target_5′ GGCAUGCAGGGAGCCAGGCA	
	miRNA_3′ UCAAGUUCUUUCGACACCUU	
**ath-miR396a**	||.|||||o|||||||||o|	
	target_5′ AGAUCAAGGAAGCUGUGGGA	
	miRNA_3′ ACACUUAGAAUUACCACGACG	
**bra-miR172b**	||||||| ||||||||o|||||	
	target_5′ UGUGAAUGGUAAUGGUGUUGC	
	miRNA_3′ GCCGUUCAGUAGGAACCGAGG	
**mtr-miR169e**	o|| ||||||||||||||||.|	
	target_5′ UGGGAAGUCAUCCUUGGCUCA	
	miRNA_3′ UCCGUUCAGUAGGAACCGAU	
**ath-miR169h**	|||.|||||||||||||||o	
	target_5′ AGGGAAGUCAUCCUUGGCUG	
	miRNA_3′ UACAUCGUAGUAGUUCUAAGU	
**vvi-miR172a**	|||.|||||||||||||||||	
	target_5′ AUGGAGCAUCAUCAAGAUUCA	
	miRNA_3′ CUACUGUCUUCGAAUCUCUCG	
**ahy-miR156b-3p**	||.||.|||||||||||||||	
	target_5′ GAGGAAAGAAGCUUAGAGAGC	

The periods indicate mismatches at the seed region (2–8mer), while the circles indicate G:U wobble.

Previous studies have also reported that the targets of miRNAs are TFs [31], which themselves regulate numerous additional genes [51]. As indicated earlier, degradome analysis of miRNAs modulated during *P. brassicae* disease development revealed that these miRNAs display a striking propensity to target TFs (Table 1). These targets include members of the following families of gene regulatory elements: *NAC* domain proteins (comprise of *NO APICAL MERISTEM* (*NAM*), *Arabidopsis thaliana* transcription factor *(ATAF1*/*2*) and *CUP-SHAPED COTYLEDON* 1, *2* (*CUC 1, 2*), Zinc finger proteins, CCAAT binding, basic helix turn loop helix (*bHLH*), MADS box, F box family proteins, Myeloblastosis (MYBs), Auxin Response Factors (ARFs) and APETALA 2 (AP2). These TFs regulate the expression of various genes and hormone homeostasis during plant growth, development and stress responses (Table 1). The role of hormone homeostasis (increased/decreased phytohormone levels) during clubroot disease progression is also well documented [5], [15]. Specifically, auxin and GH3-family of proteins (auxin-responsive/regulator) have been shown to regulate the root development in plants [19]. Therefore, these TFs possibly have a role in the regulation of clubroot development through the modulation of hormone homeostasis.

The accumulation of several miRNAs such as ath-miR156, ath-miR160, zma-miR166 and ath-miR396 during clubroot development, could modulate the root architecture and hormone homeostasis, since they are known to be involved in the regulation of the transcripts like *AP2*, *ARFs* (*ARF10*, *ARF17*) *NAC*, and a type of F-box protein (T1R1). *NAC* TFs, for example, transduce auxin signals downstream of TIR1 to promote lateral root development [52]. ARFs regulate the expression of auxin-inducible genes by binding to auxin responsive promoters (ARPs) [53]. Down-regulation of miR396 during *P. brassicae* infection of *B. napus* could have a potential impact on clubroot development since one of the possible targets of ath-miR396 is TIR1, a known regulator of auxin signaling in response to biotic and abiotic stress [52].

Another miRNA identified in our studies as being modulated during clubroot disease progression is ath-miR172. Interestingly, ath-miR172, which was highly down-regulated at 20 dpi, is known to not only interact with the pathogenesis-responsive, RAP2.7 member of the AP2 family, but also targets five other members of the same TF family [49]. In addition, previous reports [54] have demonstrated transient (1–3 dpi) expression profile changes of gma-miR168 and gma-miR172 followed by gradual decreased (12 dpi) in *Bradyrhizobium japonicum* infected soybean roots.

Furthermore, the downregulation of ath-miR169 a/b/c was reported [55] in wild-type *Arabidopsis* due to drought stress, while transgenic *Arabidopsis* plants overexpressing ath-miR169a showed enhanced leaf water loss and susceptibility to drought. In contrast, the miR169 family was increased in rice (*Oryza sativa*) during drought stress [56], and the overexpression of miR169 (sly-miR169c) in transgenic tomato led to decreased transpiration rate and enhanced drought tolerance [57]. ath-miR169 is known to target mRNAs of genes that encode members of CCAAT binding TF, as well as allowing the expression of Nuclear Factor Y (*NFY*) [58], which has important implications in stress responses [55].

The abundance of zma-miR166 and aqc-miR160 was observed to increase at 20 dpi (Table 2b, c). ath-miR160 is known to post-transcriptionally regulate TFs involved in lateral root development in *Arabidopsis* [59]. Our results are further supported by the small RNA-expression profiling of *Arabidopsis* leaves collected at 1 and 3 (dpi) with *Pseudomonas syringae* [44], which identified ath-miR160 as being highly induced. In addition to its role in lateral root development, ath-miR160 is also involved in the regulation of the TFs involved in auxin response signaling and targets ARFs: *ARF10, ARF16*, and *ARF17*, which are involved in root development in *A. thaliana* [60]. It is known that *ARF8* and *ARF17* regulate the transcription level of *GH3*-like genes, which in turn, regulates auxin level in plants [61], [62]. Free auxins or their conjugates have been shown to play crucial roles during clubroot disease development in *B. napus* [19]. Furthermore, it has been demonstrated that *A. thaliana* ARFs, *ARF10* and *ARF16*, targeted by ath-miR160, control root cap cell formation [53]. ARF mutant lines and ath-mir160-overexpressing lines showed the root tip defect with uncontrolled cell division and blocked cell differentiation in the root distal region. This resulted in a tumor-like root apex and loss of gravity sensing [53], and resembles clubroot disease phenotype of canola. Similarly, ath-miR166, which post-transcriptionally regulates *HD-ZIP III* genes, is also involved in lateral root development in *Arabidopsis* [39]. *In-situ* expression analysis has revealed that these target genes are spatially co-expressed with mtr-miR166 in vascular bundle and in the apical region of roots [40]. The over expression of mtr-miR166 has been shown to reduce the number of symbiotic nodules and lateral root development in *Medicago truncatula* [40]. In addition, ath-miR164 targets the TF NAC domain containing proteins (NAC/ATAF/CUC1), which regulates auxin signaling during lateral root development, and is also up-regulated during the *P. brassicae* disease progression and development in *A. thaliana* [28]. These results provide additional evidence for the important roles played by ath-miR160 and mtr-miR166 during root development and clubroot disease progression.

Moreover, the expression of many TFs was observed to be modulated in response to *P. brassicae* inoculation. These TFs belong to several major families, including ARF, AP2, MYB, Basic-Helix-Loop-Helix, homeobox, and zinc-finger family proteins. Also, it has been reported earlier that some of these TFs are involved in hormone homeostasis. For example, the modulation of cytokinin [15] and auxin [62] during clubroot development has previously been reported. As well, the involvement of auxins in the development of clubroot disease is also well documented [20], [21]. Furthermore, consistent with our microarray results carried out on *Brassica napus* infected with clubroot disease, transgenic *Arabidopsis* plants with lower cytokinin levels were found to be more tolerant to clubroot [15]. All of these TFs, therefore, may conceivably have a bearing on the development of clubroot disease in canola. Additional work to confirm these hypotheses through silencing of the target genes and determining their effect on the clubroot initiation and progression to establish what role they may play in pathogenesis in *B. napus* is currently underway in our laboratory.

### Relative Quantification of miRNA using qRT-PCR

Stem-loop qRT-PCR is a reliable and established method of detection and measurement of expression level of miRNAs. The stem-loop primer increases the sensitivity of reaction such that this method can significantly distinguish between two miRNAs with only a single nucleotide change [63], [64]. We used stem–loop RT followed by TaqMan (Applied Biosystems, USA) PCR analysis to validate and measure the expression of a selected sub-set of ten differentially expressed miRNAs from the ones identified in the miRNA microarray analysis as indicated previously. Endogenous controls, snoR66 was used as references at 10 dpi and 20 dpi, along with non-template controls in each set of experiments.

**Figure 3:**
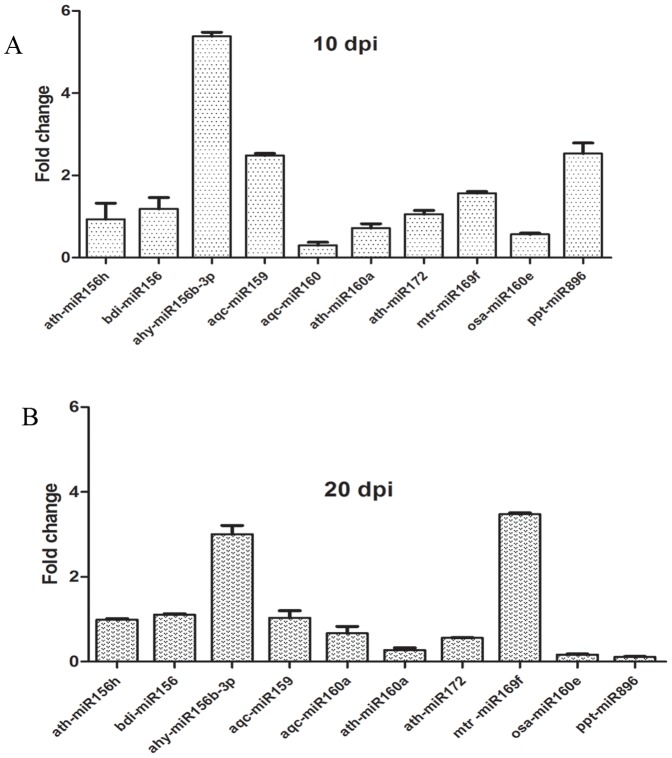
Relative abundance of miRNA in *B. napus* plant infected with *P. brassicae*. (a) Relative accumulation of miRNA showing the quantitative expression at 10 dpi and (b) Relative accumulation of miRNA showing the quantitative expression at 20 dpi. The expression of sonR66 was used as internal control in the experiment. The error bars show the standard deviation.

qRT-PCR results indicated that at 10 dpi, the expression of four out of ten miRNAs (ahy-miR156b-3p, aqc-miR159, mtr-miR169f and ppt-miR896) increased (Figure 3A), while the expression of aqc-miR160, ath-miR160a and osa-miR160e decreased (Figure 3A). At 20 dpi, two miRNAs (ahy-miR156b-3p and mtr-miR169f) exhibited increased expression (Figure 3B), while five miRNAs (aqc-miR160a, ath-miR160a, ath-miR172, osa-miR160e and ppt-miR896) showed decreased expression (Figure 3C).

Although some of the miRNAs showed similar expression patterns in both microarray and qRT-PCR experiments, this was not true in all cases. Similar non-correlation between microarray and qRT-PCR has previously been reported [65]. Indeed, a systematic analysis of different platforms of miRNA expression concluded that although the intra-platform correlation was very high, the same did not apply for inter-platform comparisons [66].

### Genome Wide Mapping of mRNA Target Cleavage Site

**Figure 4:**
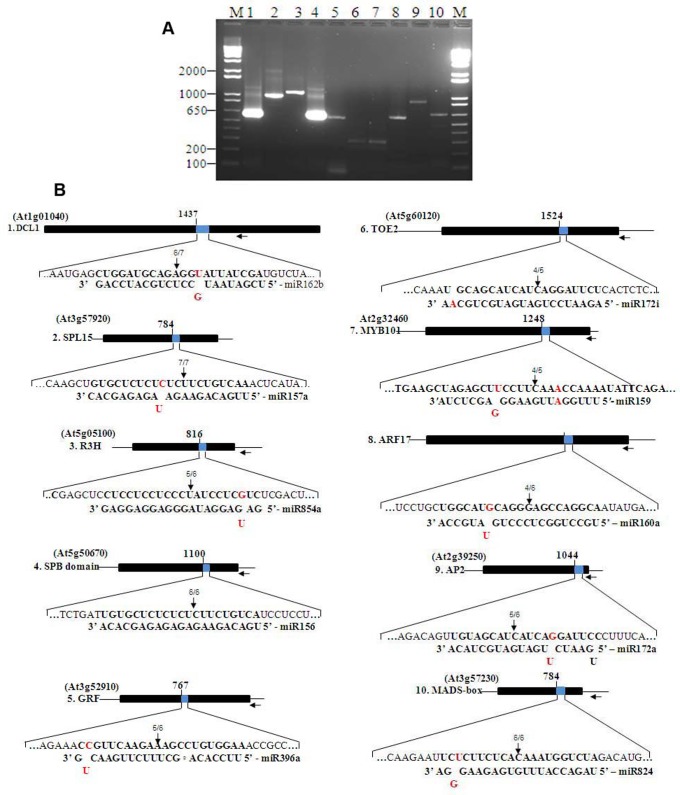
miRNA mapping and cleavage site determination through 5′ RLM RACE. Agarose gel image of 5′ RACE products (A) and the target mRNA cleavage sites (B). The targeted mRNA section and miRNA sequences, along with mismatch (es), if any, are shown as the expanded region. The 5′ends of the cleaved product determined by sequencing is indicated by the vertical arrowheads, along with the numbers of clones analyzed. The horizontal arrowheads indicated the gene-specific primer sites used for 5′RLM-RACE.

miRNA:mRNA interaction was analyzed through 5′-RLM RACE of ten selected sets to elucidate putative targets. 5′-RLM RACE generated products between 200 to 1000 bp and the major PCR products of predicted size resulting from miRNA-guided cleavage event were determined through mapping (Figure 4). These products were cloned, and subsequently sequenced. The results of 5′RLM-RACE revealed that out of 10 miRNA:mRNA interactions, six showed G:U base pairs or mismatches with no consistency in the position of the seed site. It has been previously reported that single G:U base-pair mismatch diminish the expression of the target genes [50]. The second significant feature here is that most of the mismatches observed in our case involved a single base and at a position between 2–8, and any mismatches between positions 2–8 has previously been reported to strongly reduce the expression of target genes [50]. Mostly the 5′ ends of target genes terminated at a position corresponding to the middle of the region of complementarity with respective to their miRNA and directed the cleavage (Figure 4A). This is not surprising given the fact that the aforementioned feature is the characteristic of RISC-catalyzed cleavage events [67]. The sequence analyses of the 5′-RLM RACE products thus indicate the potential mRNA targets and the miRNA-mRNA interaction sites. The results of 5′ RLM-RACE (Figure 4B) were also in agreement with the findings of degradome analysis, where most of the tested miRNAs indicated TFs as being the major targets. For example, ath-miR157 binds to the mRNA of Squamosa binding like protein (*SPL15*) regulating plant growth and development along with abiotic and biotic stress signaling [68]. Similarly, ath-miR824 is involved in the regulation of mRNA of MADS-box protein (AGL16-II) while ath-miR172 targets the mRNA of *TOE2*, which belongs to pathogenesis related AP2 like ethylene responsive factors. Similarly, ath-miR159 binds to the mRNA of *MYB101* and ath-miR160 post-transcriptionally regulate the expression of *ARF17*. *ARF17* is known to be involved in adventitious root development [62] and auxin mediated signaling pathways in plants [26]. On the other hand, ath-miR162 targets the *A. thaliana* endoribonuclease such as Dicer Like Protein 1 (DCL1) [69], which is a RNA helicase involved in the miRNA processing, having important implications in plant growth and development [70]. The current results of miRNA mapping and cleavage site determination through 5′RLM RACE suggest that *DCL1, MYB, AP2,* MADS-box, *ARF* and *TOE* are major target gene for the miRNAs (miR162, miR160, miR157, miR824, miR396, miR854 and miR172.) involved in the disease modulation and progression of clubroot.

## Conclusion

Clubroot disease of *Brassicaceae* family has potential to substantially limit canola production. A miRNA microarray-based approach was used to identify miRNA expression and regulation during the disease initiation and progression. Results from this study provide a useful basis towards understanding the dynamics of miRNAs related to *P. brassicae* infection of canola. The differential expression analysis of miRNA during pathogenesis of *P. brassicae* was carried out at two developmentally distinct time points. The expression of various miRNAs was observed to be modulated during the process of clubroot initiation and development. Many of these miRNAs were involved in the regulation of gene activity by targeting, among others, TFs, *ARFs, MYB*, MADS box, *AP2* and ERFs. Based on available literature, many of these are known to be involved in stress-responses and developmental events related to pathogenesis.

The results documented here lead us to conclude that a diverse array of miRNAs and their target genes are modulated during the pathogenesis of canola by *P. brassicae*. The current study enhances our understanding related to the biological processes involved in *P. brassicae*-canola interactions and may lead to unique ways to generate disease resistant phenotypes.

## Materials and Methods

### Plant Growth and Pathogen

Seeds of *Brassica napus* were germinated on moist filter paper in petri dishes and placed under a light/dark (16 h/8 h) regime for 7 days at 22±1°C. Clubroot gall pathotype SACAN03-1 (St. Albert, Canada type 03-1), was isolated from infected tissues stored at −20°C (collected from Southern Alberta region), [71]. Resting spores were extracted by homogenizing mature clubroot galls of Chinese cabbage, followed by filtration through six layers of cheesecloth and two subsequent centrifugation steps (2,500×g) for 10 min [72]. The resting spores of *P. brassicae* were resuspended in autoclaved, deionized water and the number of spores in the suspension was counted using a haemocytometer. One week-old seedlings were individually dipped into the spore suspensions (1×10^7^ spores/mL) for 1–2 seconds and planted in flats (3×3 cm, one seed per insert) containing LA4 Aggregate Plus (Sunshine Professional Peat-Lite Mix; Sungro Horticulture, Vancouver, BC, Canada). Plants were placed in a growth chamber with an 18 h photoperiod (light intensity of 130****µmol m^−2^ s^−1^) with a day (21±1°C)/night (18±1°C) temperature cycle for 1 week, with a water tray underneath.

### Sampling and RNA Isolation

One-week old seedlings of *B. napus* cv. Westar were divided into two groups; one was inoculated with *P. brassicae* while the other, uninoculated group, served as controls in the experiment. Tissues were harvested at 10 and 20-days-post inoculation (dpi) for RNA isolation. Plant roots from these groups were pooled separately and total RNA was isolated using the TRI-Reagent (Ambion, USA) and used in microarray experiments.

### Microscopy of *B. napus* Infected with *P. brassicae* at Two Time Points

Pathogen-inoculated roots of *B. napus* cv Westar, as well as uninoculated controls, were cut into small (10–15 mm) segments and fixed in FAA (formalin, acetic acid and ethyl alcohol, [73] under vacuum at room temperature overnight. Following fixation, the root segments were dehydrated in a series of graded ethanol/water solutions, changed to toluene and later infiltrated with Paraplast® using a Leica TP1020 Tissue Processor. Longitudinal sections (6 µm thickness) were prepared using an AO Rotary microtome, affixed to the glass slides, de-paraffinated with toluene, rehydrated through a graded ethanol series and stained with Harris Hematoxylin and counterstained with Eosin Y [74]. Slides were subsequently dehydrated in ethanol followed by toluene and mounted with DPX® mounting medium (Sigma Aldrich, USA). The sections were viewed with a Zeiss Axioskop, analyzed using AxioVision™ software and photographed with Zeiss Axiocam.

### µParaflo™ miRNA Microarray

The miRNA microarray experiment was performed at LC Sciences, Houston, Texas (http://www.lcsciences.com). Chip hybridization experiments were carried out in duplicate using total RNA samples (2 to 5 µg) which were size fractionated using a YM-100 Microcon centrifugal filter (Millipore, USA). The small RNAs were polyadenylated (3′) using poly (A) polymerase (Applied Biosystems, USA). An oligonucleotide tag (Cy3 or Cy5, for individual treatments) was then ligated to the polyadenylated small RNA for fluorescent dye staining. Hybridization was performed overnight on µParaflo microfluidic chips (MRA-1038B) using a micro-circulation pump (Atactic Technologies, USA) where each detection probe consisted of a chemically modified nucleotide-coding segment complementary to known target plant microRNA from PMRD (Plant microRNA Database; (www.bioinformatics.cau.edu.cn/PMRD) and miRdata base 15 was used. This array includes over 5600 probes from experimentally as well as computationally identified plant microRNAs mainly from *Arabidopsis thaliana*, *Brassica oleracea*, *B. rapa* and *B. napus*, *Oryza sativa*, *Populus trichocarpa, Physcomitrella patens, Glycine max, Zea mays, Vitis vinifera* and *Triticum aestivum*. Blank cells and other non-homologous nucleic acid probes served as negative controls. Hybridization was performed overnight on a single-channel µParaFlo microfluidic chip at 34°C as described by [32]. Data were analyzed by subtracting the background and normalizing the signals using a LOWESS filter (locally weighted regression). The ratio of the two sets of detected signals (log2) and *P*-values were calculated through t-test. Differentially detected signals are reported for those with *P*-values <0.05. A transcript was considered as detectable when the signal intensity was higher than 3×the background standard deviation and the spot CV was <0.5, where CV was calculated as standard deviation/signal intensity. The miRNA-microarray data have been submitted to public repository, Gene Expression Omnibus/NCBI database (accession number-GSE51590).

### Quantitative Real-time PCR Assay

Total RNA was isolated from the roots of *B. napus* treated with *P. brassicae* at 10 dpi and 20 dpi as described above. To compare the expression levels of miRNA in *B. napus* following infection with *P. brassicae*, the TaqMan microRNA Assay for quantitative Real-Time PCR (q-RT-PCR) was employed. The stem-loop primers were designed and synthesized (Life technologies, USA). In order to validate the miRNA microarray results, real time quantitative PCR using stem-loop primers [75] was carried out to investigate the differential expression pattern of ten selected miRNAs at 10 and 20 dpi which included ath-miRNA 156h, bdi-miR156, ahy-miR156b-3p, aqc-miR159, aqc-miR160a, ath-miR160a, ath-miR172, mtr-miR169f, osa-miRNA160e and ppt-miR896. For controls, we employed the *Arabidopsis* endogenous control snoR66 (Life technologies, USA). All reactions were performed in triplicate and with an additional non-template control. Quantification of miRNA expression was performed in terms of comparative threshold cycle (C_T_) with the 2^−ΔΔC^_T_ method [76].

### starBase Degradome Analysis

Plant miRNA targets were identified through computational analysis using Degradome analysis, a web based software, starBase: a database for exploring microRNA:mRNA interaction maps [48]. This analysis predicted the target mRNAs for the *P. brassicae*-responsive miRNAs. The starBase Degradome analysis program reports all potential sequences, with mismatches no more than specified for each mismatch type [48]. The minimal score among all 20-mers did not exceed 2.5 penalty score and cleavage tags >1 with default parameters.

### Mapping of mRNA Cleavage Site

To examine the miRNA-directed cleavage of their predicted targets *in vivo*, we isolated total RNA using Tri-reagent as described above from *A. thaliana* (ecotype Ws). We employed the Firstchoice® 5′ RLM –RACE kit (Ambion, USA) to amplify the 5′ UTR of the full-length target genes. These target genes were selected on the basis of their expression of their corresponding miRNAs. These miRNAs were differentially expressed or showed high level of abundance at both the time points (10 and 20 dpi) due to infection of *B. napus* with *P. brassicae*. As well, based on available literature, their targets are implicated in root/clubroot development and/or signaling pathways related to disease development/tolerance. Combinations of three different primers were sued in 5′ RLM –RACE experiment as listed in table-S1. Total RNA (10 µg) was first treated with Calf Intestine Phosphatase (CIP) to remove the free 5′ phosphate group from partially degraded RNA, ribosomal RNA, fragmented RNA, tRNA and contaminating genomic DNA. The RNA was then treated with Tobacco Acid pyrophosphate (TAP) to remove the 5′ capping from full length mRNA, which are not affected by CIP, and to generate a 5′ monophosphate. RNA was then ligated with an RNA adaptor (45 oligonucleotide-long) using T4 RNA ligase. Using the above as template in RT-PCR, we amplified the 5′ ends of mRNA using miRNA target specific primer and adapter primer. The ampilcons were then cloned into TA cloning vector, pGEMT-easy vector (Promega, USA) and sequenced (ABI prism, Applied Biosystems, USA).

## Supporting Information

Figure S1 Visualization of the miRNA microarray data through clustering heat maps showing t-test of selected *B. napus* miRNA differentially expressed following infection by *P. brassicae* at 10- dpi. Red indicates an increase in abundance, while green represents a decrease in abundance of miRNAs at a P value of less than 0.01 (P<0.05).
(TIF)

Figure S2 Visualization of the miRNA microarray data through clustering heat maps showing t-test of selected *B. napus* miRNA differentially expressed following infection by *P. brassicae* at 20- dpi. Red indicates an increase in abundance, while green represents a decrease in abundance of miRNAs at a P value of less than 0.01 (P<0.05).
(TIF)

Table S1 List of primers used in 5′ RLM-RACE.
(DOCX)
