# Supplementary figures and images for: Differential Expression of miRNAs in Brassica napus Root following Infection with Plasmodiophora brassicae
Source: PLoS One. 2014 Jan 31;9(1):e86648. doi: 10.1371/journal.pone.0086648 (PMC3909011; doi:10.1371/journal.pone.0086648)

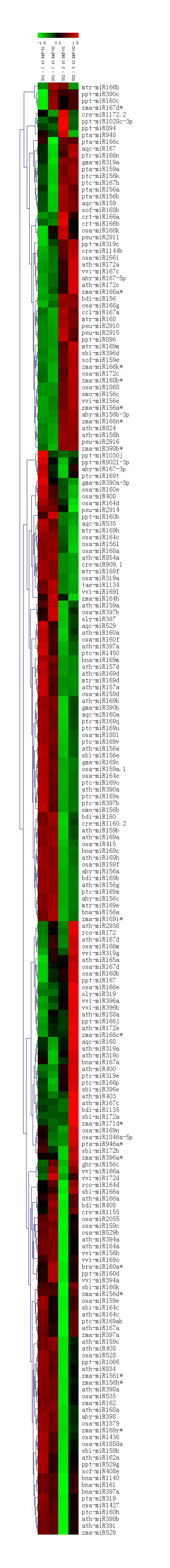

Supplement: Figure S1 — Visualization of the miRNA microarray data through clustering heat maps showing t-test of selected B. napus miRNA differentially expressed following infection by P. brassicae at 10- dpi. Red indicates an increase in abundance, while green represents a decrease in abundance of miRNAs at a P value of less than 0.01 (P<0.05). (TIF) [file pone.0086648.s001.tif]

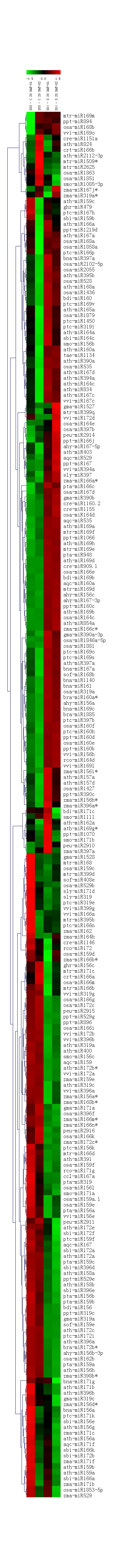

Supplement: Figure S2 — Visualization of the miRNA microarray data through clustering heat maps showing t-test of selected B. napus miRNA differentially expressed following infection by P. brassicae at 20- dpi. Red indicates an increase in abundance, while green represents a decrease in abundance of miRNAs at a P value of less than 0.01 (P<0.05). (TIF) [file pone.0086648.s002.tif]
