# Supplementary material for: Differential Expression of miRNAs in Brassica napus Root following Infection with Plasmodiophora brassicae
Source: PLoS One. 2014 Jan 31;9(1):e86648. doi: 10.1371/journal.pone.0086648 (PMC3909011; doi:10.1371/journal.pone.0086648)
Supplement: Table S1 — List of primers used in 5′ RLM-RACE. (DOCX) [file pone.0086648.s003.docx]

**Table S1.** List of primers used in 5´ RLM-RACE

| **Name** | **PRIMER** |
| --- | --- |
| CR157a_F | AGAAGTTGTCGCAGAAGACTCGCT |
| CR157a_IR | TCGAGTCGAAACCAGAAGATGGTC |
| CR157a_OR | ATCTGCTGGCTCCGAGATTTGACT |
| CR169b_F | ACAAGCATGCAATGAGAAGGGCAC |
| CR169b_IR | CACCTAACATAACTCGCTCTTTGTAC |
| CR169b_OR | CACTTTGTGGTCAGAAAGGCAAACG |
| CR156_F | TGTAGCCGCCAATGGTTCGAGTTA |
| CR156_OR | TCCATCAGAGACTGCACTCGCATT |
| CR156_IR | TGAGGGAGAAGAGAAGAGACACA |
| CR396a_F | ACATGTTGGCTGGTGCTTCTGTTC |
| CR396a_IR | TCCATGACCAAGCTCCTCACCTAA |
| CR396a_OR | AGTCTGATGATCGTGGCCAATCGT |
| CR159e_F | TCCTCGAAGCGACTTTGGGAATCT |
| CR159e_IR | TTGGTGGTGGTGGAGACTGAATGT |
| CR159e_OR | TGGAATAGTTGAAGCGCAGCTCCT |
| CR160a_F | CTCGGATCACATGGTTTCAAGGCA |
| CR160a_IR | TATCTGATGGCGGACTGCCAAAGT |
| CR160a_OR | ACTGTGTCTCATTGTCGCTGGACT |
| CR172a_F | AGTGCAAGCTTAGCAAGAGGAGGT |
| CR172a_IR | AGTCACATAATCATTCCGTCATTCAATAG |
| CR172i_F | AGCTCGACCTCAACTTGGGAATCT |
| CR172i_IR | TTGACGAAGGGCTGAGAAGGATGA |
| CR172i_OR | AAGGAGGAAGCTATGGTGGTGGTT |
| CR854a _F | TAGAAGGTGGTGGTGTTGATGGCT |
| CR854a _IR | AAGTCCGCCCTGAGTCTCTGATAA |
| CR854a _OR | ATGGAACGGAAGCGGAATCGGTAT |
| CR824_F | CTTGAATTGAGCCTTCGTGGCGTT |
| CR824_IR | AACGCCACGAAGGCTCAATTCAAG |
| CR824_OR | GCTTAAGCGCCAAACCAAAGGTCT |
| CR162b _F | ACCGGACAAAGGAAGTGGTCAAGA |
| CR162b _IR | ATGGTGTCGTGGAGGGTTCAACAT |
| CR162b _OR | ATACATCGGGACGAGCTCTCTGAA |

*(F-Forward, IR-Inner Reverse, OR- Outer Reverse)
